# Involvement of consumers in studies run by the Medical Research Council Clinical Trials Unit: Results of a survey

**DOI:** 10.1186/1745-6215-13-9

**Published:** 2012-01-13

**Authors:** Claire L Vale, Lindsay C Thompson, Claire Murphy, Silvia Forcat, Bec Hanley

**Affiliations:** 1 MRC Clinical Trials Unit, London, UK

**Keywords:** Public and patient involvement, consumer involvement, clinical trials, systematic reviews, RCTs

## Abstract

**Background:**

We aimed to establish levels of consumer involvement in randomised controlled trials (RCTs), meta-analyses and other studies carried out by the UK Medical Research Council (MRC) Clinical Trials Unit across the range of research programs, predominantly in cancer and HIV.

**Methods:**

Staff responsible for studies that were included in a Unit Progress Report (MRC CTU, April 2009) were asked to complete a semi-structured questionnaire survey regarding consumer involvement. This was defined as active involvement of consumers as partners in the research process and not as subjects of that research. The electronic questionnaires combined open and closed questions, intended to capture quantitative and qualitative information on whether studies had involved consumers; types of activities undertaken; recruitment and support; advantages and disadvantages of involvement and its perceived impact on aspects of the research.

**Results:**

Between October 2009 and April 2010, 138 completed questionnaires (86%) were returned. Studies had been conducted over a 20 year period from 1989, and around half were in cancer; 30% in HIV and 20% were in other disease areas including arthritis, tuberculosis and blood transfusion medicine. Forty-three studies (31%) had some consumer involvement, most commonly as members of trial management groups (TMG) [88%]. A number of positive impacts on both the research and the researcher were identified. Researchers generally felt involvement was worthwhile and some felt that consumer involvement had improved the credibility of the research. Benefits in design and quality, trial recruitment, dissemination and decision making were also perceived. Researchers felt they learned from consumer involvement, albeit that there were some barriers.

**Conclusions:**

Whilst most researchers identified benefits of involving consumers, most of studies included in the survey had no involvement. Information from this survey will inform the development of a unit policy on consumer involvement, to guide future research conducted within the MRC Clinical Trials Unit and beyond.

## Background

The concept of consumer involvement in clinical research is not a new one. In 1998 the UK Medical Research Council (MRC) published guidelines on good practice in clinical trials [1] which recommended consumer involvement, specifically within clinical trial steering committees (TSC) and to assist in the development of patient information material. Two UK Department of Health publications regarding clinical research within the NHS in England [2,3] recognised the value of consumer involvement, stating that patients and the public must be involved in all stages of the research process. Internationally, a number of initiatives that support consumer involvement in healthcare research have been established, for example the US National Institutes for Health (NIH) Directors Council of Public Representatives and the Cochrane Collaboration Consumer Network. Such initiatives have improved awareness and potentially led to an increased acceptance of involvement by the research community.

In the UK, surveys have been conducted to gauge the extent and type of participation of consumers in UK National Health Service (NHS) research [4] and UK clinical trials units that were conducting randomised controlled trials [5]. In this second survey, researchers contacted 103 clinical trials units in the UK. Of the 62 eligible responses received, 23 units reported that consumers had already been involved in their work. Most were positive about this involvement. 17 units planned to involve consumers. 15 centres had no plans to involve consumers, but only four of these considered such involvement irrelevant. Trials units were then contacted to seek further information about involvement in 48 individual trials. Again, responses were mostly positive. Consumers were reported to have helped refine research questions, improve the quality of patient information, and make the trial more relevant to the needs of patients.

The MRC Clinical Trials Unit (CTU) designs, runs, analyses and reports high quality randomised controlled trials (RCTs), meta-analyses and other clinical studies in a variety of healthcare areas, primarily cancer, HIV and other infectious diseases. In 2008, a Consumer Involvement Group was established at the CTU to support involvement across the breadth of its research. The Group, members of which are all employees of the MRC CTU and include a statistician, trial management staff and a systematic reviewer, was aware of some research areas and individual studies in which there had been considerable consumer involvement. However it was felt that there was a lack of consistency in the approach to involvement between research programmes in different disease areas and study types within the CTU. Therefore the Group set out to more formally assess both past and current levels of involvement across all CTU research studies, with the primary aims of:

(1) Establishing the extent of consumer involvement throughout the CTU

(2) Evaluating CTU researchers perceptions of the impact of involvement

(3) Developing guidance for researchers to improve consumer involvement in clinical studies at the CTU

(4) Informing consumer involvement in clinical research more widely

## Methods

We used the term 'consumer involvement' in line with the UK National Cancer Research Institute (NCRI), since the initial work of the CTU Consumer Involvement Group was confined to the Unit's cancer trials. Our definition of consumer involvement, which was based on the INVOLVE definition of public involvement http://www.invo.org.uk/, was the active involvement of consumers (i.e. patients, carers or family members, health service users, patient representatives or members of groups or organisations that represent those affected by the condition being researched) as partners in the research process and not as subjects of that research. For example, patients or carers as members of trial management groups (TMG; see Additional File 1: Trial Management Group Information Pack); involvement of a patient organisation in the planning of a trial, or patients contributing to the development of patient information materials.

The semi-structured survey questionnaire (see Additional File 2) was developed by members of the CTU Consumer Involvement Group. It combined a mixture of open and closed questions designed to capture both quantitative and qualitative information on whether or not the studies had involved consumers; how consumers had been identified and supported; the types of activities undertaken; researchers' perspectives on the benefits and challenges of involvement and the perceived impact of consumer involvement on their research. For categorical questions, a list of possible categories was developed by the Consumer Involvement Group based on their own experiences of consumer involvement and knowledge of external examples of consumer involvement, from the medical literature or from other research units. For each categorical question, respondents were also given an 'other' option, which they were asked to specify. Prior to wider distribution, members of the Group tested the logic of the questionnaire design and data capture using either real examples from their own work or dummy data.

The sampling frame used for the survey was all CTU-led clinical research studies that had been included in CTU progress report (April 2009). The report outlined all research, predominantly RCTs, non-randomised trials and systematic reviews across all programmes of research at the CTU, carried out over a six year period (January 2003 to January 2009). The studies represented different stages of research from early development through to studies that had been completed and reported. The lead members of staff for each study (including clinicians, senior clinical trialists, statisticians, systematic reviewers and epidemiologists) most of whom were based at the CTU, were contacted by email and asked to complete the online survey (http://SurveyMonkey.com, LLC, Palo Alto, California, USA) by following a direct link within the email. Data collection took place between October 2009 and April 2010, with data from the survey captured electronically. Members of staff who did not complete the survey by the first deadline received two further requests. After this, non-responders were replaced with alternative members of staff connected with the study wherever possible. Following data collection, responses for each study were matched with the study start date, defined as the date the trial opened to recruitment or the protocol date for the systematic reviews.

The proportion of studies with consumer involvement; roles undertaken by consumers and researchers' motivations for initiating involvement were analysed using STATA 11.1. Analyses using the study start dates were also conducted to assess whether consumer involvement had changed over time and also whether there was a difference in the extent of consumer involvement between ongoing and completed research. Ongoing research was defined as research that was either in start up, actively enrolling or in follow-up. Completed research was defined as research that was published; completed but not yet published or had been stopped or suspended.

Free text answers to questions regarding the researchers' perceptions of the impact of involvement on the research; the impact of involvement on the individual researcher and on challenges or problems faced with involvement were collated into themes by individual members of the Consumer Involvement Group. Major themes were subsequently identified through discussion among the Group until consensus was reached. Categories emerging from the written text answers from responders were then identified within these major themes.

## Results

At the outset, 34 lead CTU researchers were identified as responsible for the 160 studies included in the survey. However, alternative and additional responders were identified during the data collection process such that, in April 2010, when the survey was closed, 138 (86%) completed questionnaires had been returned by 35 named responders (128 questionnaires, 93%) plus 10 further anonymous responders (7%). These studies had start dates ranging from 1989 - 2009, and included 93 RCTs (67%), and 23 systematic reviews (17%), 15 non-randomised trials (11%) and 7 epidemiological or observational studies (5%). There were similar numbers of completed (69) and ongoing (68) studies (the status of one further trial was unknown). Sixty-nine studies (50%) were in cancer; 42 (30%) were in HIV and 27 (20%) were in other disease areas including arthritis, tuberculosis and blood transfusion medicine, giving a good representation of the work of the CTU.

### Extent of Consumer Involvement at MRC CTU

Of the 138 responses, 43 (31%) studies had involved consumers and 64 studies (46%) had no involvement. For the remaining 31 studies (23%) the respondents gave no answer (23 studies) or were uncertain whether there had been involvement (8 studies). For these 8 studies, the most common reasons given were that another organisation coordinated the project and the respondent did not know the local arrangements (n = 3) or because they had only recently become involved in the study and did not know whether there had been any involvement previously (n = 2). Of the 43 studies that involved consumers, 34 (79%) were RCTs.

**Figure 1:**
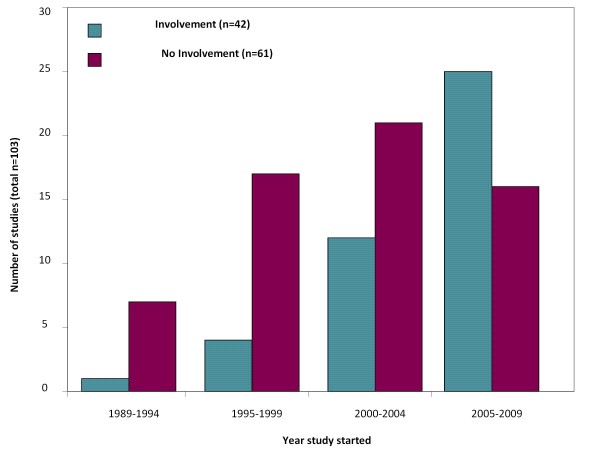
**Consumer involvement by trial start year**. Based on 103 trials (31 studies with unknown involvement status and 4 studies with unknown start dates excluded).

When studies were grouped by start dates (Figure 1), of the 8 studies that started before 1995, almost all of which are now completed (90%), only one study had consumer involvement. For the 41 studies initiated between 2005 and 2009 (73% completed), 25 (61%) had consumer involvement. Indeed all of the RCTs initiated in 2009 (n = 4) had consumer involvement. There was also a higher frequency of consumer involvement in ongoing studies (27/68, 40%) than in completed studies (16/55, 23%; p = 0.037).

### Reasons for involving consumers

**Figure 2:**
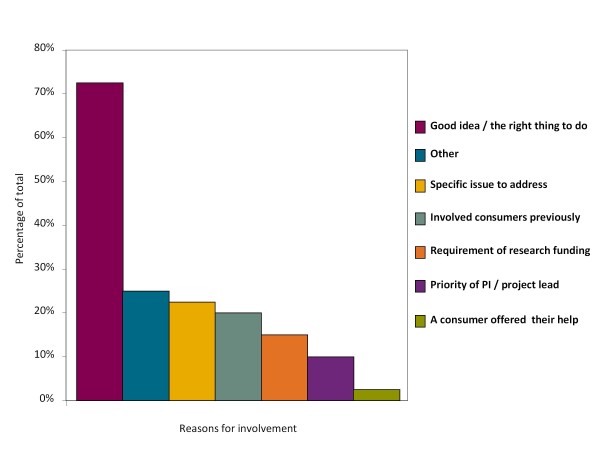
**Reasons for involving consumers**. Based on 43 studies with consumer involvement.

CTU researchers were asked to select their reasons for involving consumers (Figure 2). The most common reason selected (29 studies, 73%) was that researchers thought it was the right thing to do. Responders cited other reasons for involvement in 11 studies (26%), included learning more about the disease or population (2 studies); that involvement was essential or necessary in relation to recruitment, ensuring the appropriateness of research materials or giving guidance on issues (3 studies) and because funding bodies recommended involvement, without it being a requirement (3 studies). Three further respondents did not know what reasons had lead to involvement in their studies.

### How consumers were involved in MRC CTU studies

Researchers were asked about how many consumers had been involved in their studies, how they had been identified or recruited and what types of activities they had taken part in. Most studies involved less than three consumers (31 studies, 72%) with 14 studies involving only one consumer. However, some involved considerably more, for example, the Micobicides Development Programme (MDP) involved a large number (> 100) of patient and community representatives in focus groups to inform the study design. Overall, consumers were identified from a variety of sources, although they were often already known to a member of the research group (n = 31, 72%), for example as current or former patients of the clinical investigators, or through previous involvement in a research study. Only one study had actively attempted to recruit consumers by advertising the opportunity.

**Figure 3:**
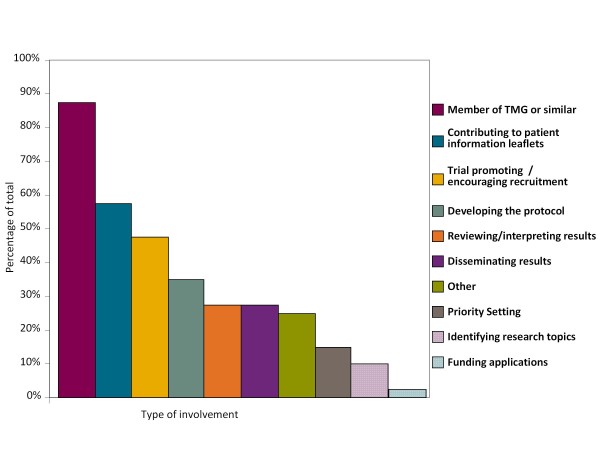
**Activities undertaken by consumers**. Based on 43 CTU studies with consumer involvement.

Consumer involvement in CTU studies was most commonly as part of a trial management group (TMG) or similar study advisory or steering group (35/43, [88%], Figure 3). Consumers in these studies undertook a variety of activities, for example, writing or commenting on patient information sheets (24 studies, 67%); trial promotion activities (19 studies, 54%); aspects of protocol development (14 studies, 40%) or interpreting and disseminating the results (9 studies, 26%).

### Perceived impacts of Consumer Involvement

Table 1 gives a summary of the responses to semi-structured questions relating to the respondents perceptions of benefits of consumer involvement; problems with involvement; the impact of involvement on the study and also on the individual researcher for those studies with consumer involvement. Whilst we had intended to analyse the results for the individual questions separately, there was significant overlap between the responses for questions relating to benefits and impacts. Therefore, themes were identified from the combined responses to these three questions (n = 90). The individual responses were then grouped within these themes.

**Table 1:** Summary of responses to semi-structured questions from 43 studies with consumer involvement

Question	Responses (n)			
	No	Yes - no reason given	Yes - reason given	Did not respond
**1. Were there any benefits of consumer involvement?**	1	2	**37**	3
**2. Were there any impacts of involvement on the research study?**	11	3	**26**	3
**3. Were there any impacts of involvement on the researcher?**	10	3	**27**	3
**4. Were there any problems of involving consumers?**	28	3	9	3

The six major categories emerging from the textual responses to these questions were:

#### i. Improvements in study design and recruitment

Researchers running studies that had involved consumers felt that patients' experiences, for example, of treatments being used in the trials, were very valuable in trial design and provided insights into why patients may (or may not) consent to a study. Researchers felt that this potentially resulted in improved acceptability of the study designs to patients considering entering the trial, which may have also impacted indirectly to improve recruitment, for example:

**TRISST**: A Trial of imaging and schedule in seminoma testis: http://www.ctu.mrc.ac.uk/research_areas/study_details.aspx?s=40 Consumers contributed to TMG discussion about recruitment, "specifically ideas as to why certain patients would consent to enter TRISST which offers different surveillance schedules to detect relapse rather than opt for adjuvant chemotherapy"

**STALWART trial **[6]: An RCT of Aldesleukin with or without anti-retroviral therapy in patients with HIV-1 Infection. Consumer involvement through a Community Advisory Board, representing international trial centres "ensured an active connection to diverse people in their local communities" and "ensured that the community's needs and concerns were expressed to researchers". This enabled researchers to better understand issues that may impact on research.

**FORTE study **[7]: A RCT evaluating treatment combinations in individuals with HIV-1 infection. "It was difficult to tell the precise impact of consumer involvement" but the researcher felt that it had "helped recruitment" and regarding changes to the trial design, found that consumers were "helpful in discussions about what would be acceptable to participants"

**PRION-1 trial **[8]: A patient preference study evaluating the activity and safety of quinacrine in human prion disease. "Consumers certainly had a lot of input into study design and reviewed the protocol" with the results that, "Trial design was far more acceptable to patients and their families and uptake was very high (~76% of eligible patients joined)"

**MDP301 study: **A randomised, double-blind study to evaluate microbicide gels for the prevention of vaginally acquired HIV infection. http://www.ctu.mrc.ac.uk/research_areas/study_details.aspx?s=16 "Consumer involvement was absolutely necessary to ensure the research materials were appropriate and to recruit people to the studies". Feedback (from consumers) meant that "clinic procedures could be streamlined, and recruitment strategies amended". Without involvement of consumers the researchers felt that "recruitment and retention targets could not have been met".

**CHAPAS-1 **[9]: A study of the pharmacokinetics and adherence to simple antiretroviral regimens for children with HIV in Africa "It helped to get the carers' perspective on the research and to ensure that all the research was appropriate and acceptable to the local population."

#### ii. Improvements in study promotion and dissemination

Researchers also felt that where consumers had been involved in various trial promotion activities, there may also have been an impact on recruitment (Box 2). For example, consumers helped to promote specific trials or educate others more generally about clinical trials through involvement in patient support groups or networks. In addition, researchers at the CTU had involved consumers in the dissemination of their research findings. For example, identifying additional routes for dissemination e.g. patient groups or networks and helping to prepare lay summaries of the scientific findings, for example:

**RADICALS**: Radiotherapy and androgen deprivation in combination after local surgery. A randomised controlled trial in prostate cancer http://www.ctu.mrc.ac.uk/research_areas/study_details.aspx?s=28 Consumer involvement provided a direct "connection to patient communities" for promotion of the trial

**PREDICT: **A randomised controlled trial of continuous positive airway pressure treatment in older people with obstructive sleep apnoea/hypopnoea syndrome http://www.ctu.mrc.ac.uk/research_areas/study_details.aspx?s=81 The consumers (who were part of the trial steering committee) helped in the development of an educational YouTube video about obstructive sleep apnoea

**MDP301 study: **A randomised, double-blind study to evaluate microbicide gels for the prevention of vaginally acquired HIV infection. http://www.ctu.mrc.ac.uk/research_areas/study_details.aspx?s=16 Consumers were involved in "Peer education about the trial, about adherence (to the study treatment) and HIV prevention in general"

**DART **[10]: Development of AntiRetroviral Therapy in Africa - A randomised trial of monitoring practice and structured treatment interruptions in the management of antiretroviral therapy in adults with HIV infection in Africa http://www.ctu.mrc.ac.uk/dart/ The researcher stated that involving consumers, "hugely aided dissemination" at the end of the trial, as well as having been important in "uptake and acceptance" at the start of the trial. They also commented that consumers had been very useful in helping to deal with some negative reactions to the trial at one point.

#### iii. Improvements in study documentation

Furthermore, involvement of consumers in studies was felt to have helped to improve the study documentation. Researchers at the CTU identified several stages of their research where consumers had this sort of impact, including protocol development, writing patient information and also involvement in writing the study papers and other documents. In one study, consumers had also been involved in a funding application, for example:

**TRISST **A Trial of imaging and schedule in seminoma testis http://www.ctu.mrc.ac.uk/research_areas/study_details.aspx?s=40 Consumer involvement was beneficial in "wording for our patient information sheet update during a major protocol amendment submitted to ethics"

**COIN **[11]: An RCT comparing continuous chemotherapy plus cetuximab, or intermittent chemotherapy with standard chemotherapy in the treatment of patients with metastatic colorectal cancer http://www.ctu.mrc.ac.uk/research_areas/study_details.aspx?s=10 The consumers "helped remind us of the patient perspective on lots of issues. They also helped us with the trial launches and with patient information - getting the patient perspective on the lay summary was really helpful"

**PRION Systematic Review **[12]: Treatments for human prion disease: a systematic review http://www.ctu.mrc.ac.uk/research_areas/study_details.aspx?s=56 "It was useful to have someone with a non-scientific perspective commenting on the manuscript, for example questioning results or processes that are often taken for granted. They were also very helpful when preparing a lay summary of the scientific findings, for dissemination to a wider audience."

**DOMINO-AD **[13]: Donepezil and Memantine in moderate to severe Alzheimer's disease http://www.ctu.mrc.ac.uk/research_areas/study_details.aspx?s=50 "The (consumer) members who sit on our committee are actively engaged with the trial and offer very useful comments on how patients/carers may view the trial and trial materials. They have also made a valuable contribution to our application for additional funding."

#### iv. Improvements in decision making

Consumers were also involved in making important decisions about the study, or in helping researchers to reach what they felt to be better decisions. For example, in one trial, consumers contributed to a decision regarding continuing the use of a particular drug in the trial design. In a systematic review, consumer input had led the research group to undertake a related research project when the review was unable to address an issue that was important to the consumers, for example:

**STAMPEDE**: Systemic Therapy in Advancing or Metastatic Prostate Cancer: Evaluation of Drug Efficacy http://www.ctu.mrc.ac.uk/research_areas/study_details.aspx?s=34 "Just before the trial launched, there were problems with one of the trial drugs; STAMPEDE was investigating celecoxib and a drug in the same family (cox-2 inhibitors), rofecoxib, had shown cardiac problems in another trial in a completely different context. All trials involving cox-2 inhibitors were stopped indefinitely. The TMG had to decide whether to drop this drug and continue with the remaining arms or to wait until there was further information about these agents. Our consumers were absolutely vital in helping us come to a good decision (which was to wait a while and keep the drug in the trial)"

**IPDMA in cervical cancer **[14]: Concomitant chemoradiotherapy versus radiotherapy alone for cervical cancer: a systematic review and meta-analysis of individual patient data. http://www.ctu.mrc.ac.uk/research_areas/study_details.aspx?s=45 "As a result of involving the consumers, we decided to become involved in a subsequent piece of research that we hoped would allow us to provide information on late treatment effects. I'm not sure we would have done so if the consumers hadn't been involved. Our consumers are also involved in helping us to decide how best to involve others in our future meta-analyses"

**N-Alive: **Can we stop heroin injectors dying from an overdose if we give them a drug called Naloxone to carry around with them when they leave prison? http://www.ctu.mrc.ac.uk/research_areas/study_details.aspx?s=80 "Focus group members helped to decide on the information inserts and the wallet to be used for dissemination of the Naloxone in the trial."

#### v. Increased confidence in the study

Consumer involvement enabled researchers to feel more confident that the studies they were conducting were targeting and responding to consumer needs. They could feel more certain that they were appropriately dealing with patients' concerns and addressing the most relevant issues by patients and their families. Researchers also indicated that working with people who had first hand experience of a disease enabled them to better understand the condition or treatments being studied and to put the research into context, for example:

**QUARTZ**: Does radiotherapy improve patient's quality of life when lung cancer has spread to the brain? http://www.ctu.mrc.ac.uk/research_areas/study_details.aspx?s=27 "I think it gave the trial much greater credence, but if I'm totally honest, I can't think of any major things that would not have happened if we had not had consumer involvement. However, the consumer was extremely positive about being involved, and spoke to many consumer groups about this."

**CHORUS**: A large randomised trial to determine the impact of timing of surgery and chemotherapy in patients with ovarian cancer. http://www.ctu.mrc.ac.uk/research_areas/study_details.aspx?s=9 The researcher reported that consumer involvement "helped endorse the relevance and credibility of the trial" and that resulted in them having "more confidence in the relevance of the research"

**MIA001 **[15]: A randomised trial to assess the safety of a vaginal microbicide gel versus vehicle placebo in Uganda http://www.ctu.mrc.ac.uk/research_areas/study_details.aspx?s=136 A researcher from the trial team felt that there had been a direct impact of involving consumers in terms of the team "being more confident that we were appropriately targeting and responding to consumer needs"

**DART **[10]: Development of AntiRetroviral Therapy in Africa - A randomised trial of monitoring practice and structured treatment interruptions in the management of antiretroviral therapy in adults with HIV infection in Africa http://www.ctu.mrc.ac.uk/dart/ The researcher felt that there had been "Huge benefits in terms of credibility and acceptance" from consumer involvement.

**PRION Systematic Review **[12]: Treatments for human prion disease: a systematic review http://www.ctu.mrc.ac.uk/research_areas/study_details.aspx?s=56 The researcher said that working with the consumer had "made me think much more about the impact of the disease and potential treatments, as well as attitudes of the scientific community towards patients and their carers, and gave me a new perspective on the work I was doing."

#### vi. Challenges for researcher in involving consumers

Although the majority of responses indicated that researchers felt positive about their experiences of involving consumers, there were some challenges. These included additional resources or time required to involve consumers; consumers becoming too unwell during the course of the research to continue their involvement or having difficulties attending meetings. Some researchers also reported that they had found it difficult to know what to expect from the consumers and did not know where to go for help or advice. Most of those who had involved consumers indicated that they had received no training on how best to involve consumers in their studies and many indicated that some kind of support (e.g. guidelines or mentoring) may have been helpful.

## Discussion

Although we surveyed more than 40 individual CTU researchers spanning 160 projects and had a high response rate (86%), we found that only 43 studies (31%) had involved consumers. This is a slight increase on previously reported rates of consumer involvement in clinical trials found in a number of previously published surveys [4,5,16] A survey of 103 UK clinical trials units in 2001 found that 23 (22%) were actively involving consumers [5] and a national postal survey of 900 randomly selected researchers in the UK in 2007 reported that 88 (17%) were involving consumers, mainly as members of project steering groups; in the design of research instruments and/or in the planning or designing of the research methods [4]. More recently, public involvement in applications made to the UK National Research Ethics Service in 2010 has been reported [16]. Of 646 applications, 95% of which described clinical trials, only 124 applications (19%) adequately demonstrated ongoing or planned public involvement. For those applications with involvement, in keeping with the results from our survey, members of the public undertook a variety of tasks spanning all aspects of the study, most commonly being involved in design and dissemination. Because studies in our survey spanned a 20-year period, we have also been able to demonstrate an increase in involvement at MRC CTU since the early 1990s, such that around two thirds of all studies initiated in 2009 (100% of new RCTs) had consumer involvement. The number of studies that involved consumers seems to have increased since 2005, which coincides with the first publication of the UK NHS Research Governance Framework [2] which recommended that researchers should actively involve consumers. Subsequently, in 2007, the CTU Cancer group launched a guidance document for consumer involvement in trials (see Additional File 3). Whilst our results do not tell us the reasons for increased consumer involvement in the CTU around this time, it is likely that as a consequence of the framework being published, there was a greater awareness of consumer involvement that may have had an indirect impact on CTU researchers.

Interestingly, our results show that the majority of consumers had been involved as members of committees; in particular trial management groups often as the only consumer involved in the study, which we recognise can be difficult for the individual and may not be appropriate. The CTU has worked with consumers who take part in these groups to improve their experiences. This has resulted in the production of an induction pack for new TMG consumer members (see Additional File 1), which has been very useful and may have broader application for other organisations. Our results seem to indicate that, in general, membership of such groups leads to consumers being involved in additional trial-related activities, including commenting on patient information or helping to improve recruitment into studies.

Researchers generally thought that involvement was worthwhile, perceiving benefits in trial design and quality, recruitment, dissemination and decision making. We found that it was difficult to assess the direct impact of consumer involvement on either the research or the researchers. For example, where consumers had been involved in helping to improve recruitment into a trial, it was not possible to evaluate whether any changes in recruitment were as a direct result of that input or not. A Cochrane systematic review of consumer involvement in clinical research [17] found very few examples where it had been possible to draw direct comparisons of consumer involvement with no involvement. We certainly did not identify any such comparisons being carried out within CTU research studies. An ongoing MRC funded research project http://www.mrc.ac.uk/ResearchPortfolio/Grant/Record.htm?GrantRef=G0902155&CaseId=16504 is currently aiming to contribute more robust approaches to measuring the impact of consumer involvement in clinical research. Once such methods become available they could be implemented in MRC CTU research to provide a better assessment of the impact of consumer involvement.

The specific aims of this survey meant that only the researchers, and not the consumers, were consulted. However, opinions and feedback of consumers who have participated in MRC CTU research, particularly in cancer trials, has previously been sought through workshops organised by the MRC CTU Consumer Involvement Group. Furthermore, the authors acknowledge that some aspects of the survey design may have limited the responders, for example forcing them to select from predefined categories rather than giving free text responses. For practical reasons the survey was not anonymised. This had some advantages in that it enabled targeted reminders to be sent and probably facilitated the high response rate. However, we acknowledge that some responders may have felt restricted in how they answered the questions given that their colleagues would collate and analyse the data.

Whilst our results suggest that the trend has been towards increased involvement over recent years, around one third still have no direct consumer involvement. Recently we have tried to address this by involving a consumer in the CTU Protocol Review Committee. The consumer's role is to read and comment on protocols, focussing in particular (but not exclusively) on patient information sheets and consent forms and more generally, contributing to discussions about all proposals, especially from the perspective of patients and/or potential participants. This ensures that all new trial protocols have some consumer input prior to the trial being finalised. Moreover, researchers identified some barriers, including additional time and resources needed and problems when consumers were unwell. We now aim to identify key areas of support that researchers at the CTU need, which we hope will guide them to effectively involve consumers in future work. As a first step we have recently updated guidance documents for trial staff (Additional Files 1 and 3) which may be helpful more broadly to those considering consumer involvement in clinical trials and have recently been involved in the development of guidance for researchers on patient involvement in clinical trials for INVOLVE http://www.invo.org.uk. We also aim to develop a CTU policy on consumer involvement for future studies and will continue to monitor involvement across the CTU. We envisage that consumer involvement in the program of clinical trials and other clinical studies at CTU will be the focus of this activity, with priority given to those trials where involvement is likely to have the greatest impact, for example those with patient centred outcomes, potentially complex treatment schedules, interventions that have had wide media coverage etc. We hope that these measures will ensure that consumer involvement at the CTU continues to improve and develop in our ongoing and future research

## Conclusions

Results from this survey have provided some evidence as to the extent and nature of consumer involvement at a large clinical trials unit. Whilst involvement has increased over time, further improvements are necessary. We would encourage those involved in the conduct of clinical research to share their experiences of consumer involvement to further expand the evidence base in respect to impacts of involvement and so that others may learn from them. We have also attempted to identify the ways in which this involvement is seen to have benefited the research process. However, more robust tools are needed to help researchers to assess the impact of involvement routinely and prospectively in their studies.

## Conflict of interests statement

The authors declare that they have no competing interests.

## Authors' contributions

CV had the original idea of conducting this survey. All authors were involved in its design and development. CV set up the electronic survey and administered the data collection. LT and CV analysed the quantitative data. All authors analysed the qualitative data. CV drafted the manuscript with input and comments from all authors. All authors read and approved the final manuscript.

## Authors' information

All authors are current members of the MRC CTU consumer involvement group. CV chairs the Group and is a member of the Meta-analysis group at the MRC Clinical Trials Unit. She has experience in conducting systematic reviews in cancer, recently having involved a group of patients in a review of cervical cancer treatments. LT is a trial statistician in the Cancer Group at the MRC CTU and has been involved in the Consumer Involvement Group for a number of years. CM is a trial manager for trials in prostate cancer also at the MRC CTU, and has worked with consumers in a number of the trials in which she is involved. SF is a trial manager for paediatric HIV trials at MRC CTU. BH is an independent communications consultant at the MRC CTU, and has many years experience in working on consumer involvement in health research.

## Supplementary Material

Additional file 1 Trial Management Group Induction Pack Click here for file

Additional file 2 Semi-structured Survey Click here for file

Additional file 3 Guidance document for Trial Managers Click here for file
