# Supplementary material for: Involvement of consumers in studies run by the Medical Research Council Clinical Trials Unit: Results of a survey
Source: Trials. 2012 Jan 13;13:9. doi: 10.1186/1745-6215-13-9 (PMC3398265; doi:10.1186/1745-6215-13-9)
Supplement: Additional file 1 — Trial Management Group Induction Pack [file 1745-6215-13-9-S1.DOC]

**
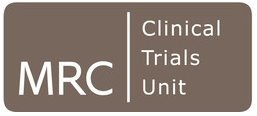
**

**Becoming a consumer member**

**of a Trial Management Group (TMG)**

**in the Medical Research Council**

**Clinical Trials Unit**

**Cancer Group**

**1. Introduction**

Thank you for considering our invitation to become a member of a Trial Management Group (TMG) in the Medical Research Council Clinical Trial Unit’s (MRC CTU) Cancer Group. This pack tells you more about TMGs and the role that you might have within a TMG. We have written it in partnership with consumers who have been involved in TMGs within the CTU. We thank them very much for their help. If there are things you think we should add to this pack, or if you have any questions, please contact Lindsay Thompson ([lct@ctu.mrc.ac.uk](mailto:lct@ctu.mrc.ac.uk); 020 7670 4622).

**1.1 About the MRC CTU (“the Unit”)**

The MRC CTU is one of the UK’s leading centres for clinical research. The Unit specialises in cancer and HIV/AIDS, but we also undertake research in other illnesses and conditions, including rheumatoid arthritis, tuberculosis and mental health. We design and run clinical trials (see section 1.3 for more information about clinical trials). We also bring together the results of a number of trials that look at the same illness or condition. This is called a systematic review or meta-analysis. We also undertake observational studies to evaluate the impact of new interventions in routine care. Our research helps to improve health care in the UK and around the world.

The Unit employs over 150 staff and is currently co-ordinating around 30 trials open to patient recruitment and many other research studies.

**1.2 About the MRC CTU Cancer Group**

The Cancer Group designs, runs and analyses trials in many different cancers. Our trials compare treatments (sometimes called “interventions”) by looking at survival, recurrence, side effects and quality of life. We also work to improve the way trials are carried out, so that we can test treatments more quickly and more reliably. We have helped to improve treatment for oesophageal, bladder, lung and kidney cancer, amongst others. With 8 to 12 open trials at any one time, the MRC CTU is the only core-funded cancer trials organisation within the Medical Research Council.

Most of our trials are large randomised controlled trials, which compare two or more treatments. Treatments being compared include chemotherapy, radiotherapy, biological agents, surgery and/or combinations of some of these. In some trials we also test non-therapeutic approaches such as different follow-up policies and schedules. Many of the trials we run are international, taking place across Europe, the USA and other parts of the world.

**1.3 About clinical trials**

Clinical trials are research studies involving patients, which compare a new or different type of treatment with the best treatment currently available (if there is one). Some clinical trials also look at possible ways to prevent illnesses, for example by testing new vaccines. No matter how promising a new drug or treatment may appear during tests in a laboratory, it must go through clinical trials before its benefits and risks can really be known.

Trials aim to find out if treatments used in health care:

- Are as safe as possible
- Have side effects (whether short or long term)
- Work better than the treatment used currently
- Help people feel better

It is now widely agreed that a properly run clinical trial is the best way to assess whether a treatment is, or is not, safe and effective.

The MRC CTU carries out clinical trials in health care. We use trials to look at the best ways to:

- Prevent illnesses, such as HIV/AIDS
- Diagnose illnesses – for example by using scans and blood tests
- Treat illnesses – for example by testing the effectiveness and safety of new drugs
- Help people control their symptoms

Clinical trials are the most reliable and best way of testing a new treatment, or of seeing whether one treatment works better than another. A new treatment is not always better, and can sometimes be less effective or less safe than existing treatments. Trials are therefore really important when we need to know whether one treatment is safer and more effective than another. We need clinical trials to improve treatment and care for patients now and in the future.

Many of the treatments now commonly used in the NHS have been tested through clinical trials. For example, in cancer care, trials have been used to try out new treatments – radiotherapy, chemotherapy, surgery, and complementary therapies. Trials have also been used to find out the best ways of using these treatments. This has meant that many people with cancer, HIV/AIDS and many other illnesses now live longer and have a better quality of life.

**More information**

You can read more about the MRC CTU, the Cancer Group and about clinical trials on our website – [www.ctu.mrc.ac.uk](http://www.ctu.mrc.ac.uk/). You can read more about patient involvement in research at [www.invo.org.uk](http://www.invo.org.uk/).

**2.** **About the** **Trial Management Group**

**2.1 What is the role of a Trial Management Group?**

Trials are developed by a Trial Development Group (TDG). The TDG will formulate the question that a trial will address, and will write the protocol for the trial. This is the set of rules about who can enter the trial and how the trial should be run, and it includes the information to be given to patients. The TDG will also apply for funding and will get to the point where a trial is launched. At this point the TDG ceases to exist, and the trial has a Trial Management Group or TMG. Usually most people who were on the TDG will become members of a TMG (for example the Chief Investigator will be on a TDG and then the TMG). Some TDGs may contain consumer representatives or may have had consumer involvement, but at the MRC CTU we want to make sure that all TMGs have consumers on them, whether or not there has been any consumer involvement beforehand.

The TMG has responsibility for the day-to-day running of a clinical trial. This includes:

- Ensuring that the trial protocol is followed
- Ensuring that the trial is run to the highest standards of ethics and of quality
- Reviewing paperwork for the trial – for example the patient information sheet, protocol, clinical report forms and so on.
- Responding to queries from any of the hospitals (or other centres) that are recruiting patients to the trial.
- Reviewing and agreeing any changes to the protocol as the trial progresses
- Promoting the trial – for example talking about the trial at meetings and conferences and writing papers about the trial.

At the end of the trial, the TMG will:

- Review the preliminary results of the trial
- Hold an investigators meeting to present the results to doctors and nurses and others who have been involved in the trial.
- Write academic papers that are written to tell people the results of the trial. These are usually written by the Chief Investigator and the project lead.
- Discuss how to disseminate the results to all interested parties, including patient organisations and/or participants (if this has not already been decided as part of the protocol)

The TMG does NOT have legal responsibility for the trial. This is the responsibility of the trial sponsor (see section 6 below).

**2.2 Who is on a TMG?**

The TMG has members to represent the perspectives of all of the stakeholders on a trial. So this will include:

- ***The Chief Investigator (CI)***

This is the lead person for the trial. He or she has responsibility for the trial, and is named on the ethics committee application. The CI is usually a senior doctor with a strong interest in the area the trial covers. He/she usually chairs TMG meetings.

- ***Doctors***

The TMG will also include a number of doctors who are specialists in the area being covered in the trial. There is usually at least one oncologist. If the trial involves surgery, there will be a surgeon. If it involves radiotherapy, the TMG will include at least one consultant clinical oncologist.

- ***Nurse***

There is sometimes a nurse on the TMG. This is important as it is often nurses who ask patients to take part in a trial and who provide care and advice to people during their cancer treatment.

- ***Pharmacists***

There is sometimes a pharmacist on the TMG. This is usually when a new drug is being tested in the trial so they can provide expert advice on the chemistry of the drug and help the TMG to monitor the safety of the patients.

- ***The Project Lead***

Within the MRC CTU Cancer Group, we have a small number of individuals who have a lot of experience of running clinical trials. They each take responsibility for overseeing a number of trials. They are called Project Leads, and the relevant Project Lead will be a member of the TMG.

- ***The Trial Manager***

The Trial Manager has the responsibility of managing the trial on a day-to-day basis. He or she is the person you are likely to have most contact with.

- ***The Data Manager***

The Data Manager is responsible for the collection of data, which is the information collected about each person who takes part in the trial. He/she also assists the Trial Manager in the day-to-day running of the trial.

- ***The Statistician***

Each trial has a statistician. He/she is responsible for working out how many people need to take part in a trial to make sure we can reach a reliable conclusion. During the trial, the statistician monitors the quality of the data collected and produces reports for the Independent Data Monitoring Committee (see section 6). When a trial is finished, the statistician analyses all the data collected.

- ***The consumer***

We aim to have at least one consumer member on all of our TMGs. We talk about your role in section 3.

**2.3 How often does the TMG meet, and for how long?**

When the trial is being planned, the trial development group (TDG) will meet every couple of months or so – although this varies from trial to trial. Once the trial is launched, the TDG will turn into a TMG and can meet about every month – although the frequency of meetings will vary between trials. Then, as the trial progresses, the meetings will happen less often. So for example, in the first two years the TMG could meet about every month. But after about two years, the TMG may meet less frequently – perhaps every six months. Then when the trial has finished and the results are nearly ready, the TMG will meet more frequently. Trials usually run over a period of five to ten years, so this can be a long commitment. People can withdraw from the TMG at any time.

Most meetings take place over the phone but there are also face-to-face meetings. Phone meetings usually last about 45 minutes. Face-to-face meetings usually last about four hours. Sometimes the trial manager may contact TMG members between meetings if they have particular queries.

**3. What’s my role as a consumer on a TMG?**

Along with other members of the TMG, you share the general role outlined in section 2.1. But there are also specific skills and experience that a consumer member of a TMG brings. Your job is to bring the patient perspective to discussions. So this might include:

- ***The patient information sheet and consent form***

As someone who has used cancer services and who may have been asked to take part in a trial, you will have a useful perspective to offer about the patient information sheet and consent form. You may be asked to help write these documents, or to comment on drafts. You may want to ask others to comment too – perhaps people from the same voluntary organisation or patient group as you.

- ***Promoting the trial***

Whilst the trial is running, you might want to write a short piece about it for a voluntary organisation or patient group newsletter or website. You might want to give a short talk about it at a patient group meeting, locally or nationally. We know that this can help to boost recruitment to trials.

- ***Helping to tell people about the results of the trial***

It is very important to get the message out about the results of a trial – not just to the clinicians and researchers, but to patients and carers. So you might want to write or comment on the draft of a feedback sheet to people who took part in the trial. You may also want to write something for a patient group newsletter or give a talk at a meeting or conference.

- ***Quality of life issues***

Most CTU trials look at the quality of life of people who are taking part in trials. Often a patient perspective can be really useful when thinking about what questions to ask trial participants about their quality of life. In one trial, the consumer member wrote a covering letter, which went on the front of the quality of life questionnaire, explaining how important it was to fill it in.

- ***Discussing the progress of the trial and any issues that arise***

Over the course of a trial many issues arise which it is helpful and important to have a consumer perspective on.

**Here is some advice from Dave Ardron, a consumer who has been actively involved in QUARTZ, a lung cancer trial at the CTU:**

*The main questions you should always ask as a consumer are:*

1. *Is the research going to benefit patients in the long term?*
2. *Is the research going to benefit patients in the short term?*
3. *If not, is there some possibility that the research will contribute to the knowledge base that will make further research a possibility?*
4. *Will the results of the trial change the way doctors do things, and possibly alter it for the better?*

*Attend as many of the meetings as you possibly can. Get to know the other group members socially as much as you possibly can. This may be difficult but it is essential. It will help you to contribute to meetings.*

*Don’t feel under pressure to contribute to meetings, but do so whenever you feel it is relevant, and remember that if you miss out on the meeting, after consideration you can contribute to the debate electronically*

*Inform yourself as much as possible about the research subject and research methodology. Read, attend conferences, and undergo training if this is available. There are some excellent opportunities for consumer training (National Cancer Research Network courses, Royal Marsden Study Days, etc).*

*Don’t be afraid to ask the “stupid” question. The researchers are sensitive to your position and will not embarrass you. They will be supportive and offer help and advice. If you do feel you are being fobbed off, seek help, from other MRC consumer reps.*

*Can you understand the patient information sheet? But more importantly, would someone with a reading age of about 10 (above the national average!) be able to? Does it fully explain the randomisation and the “we don’t know the best treatment” points?*

*Within the bounds of confidentiality seek the opinions of other patients/patient groups. You may need to ask the CI before you do this. They will usually be happy for you to do so. Do not discuss the detail of the trial with members of the general public. If in doubt, ask.*

**4. Will I be reimbursed for my expenses?**

For each meeting you attend, you will have any expenses that you incur, such as the cost of train tickets or telephone charges, reimbursed to you. You will also receive an honorarium. Please note that most teleconferences organised by the MRC CTU are free phone numbers and you will not be charged for them. You can check with the trial or data manager whether a call will be free or charged.

The Trial Manager or Data Manager will provide you with an expense form to reclaim expenses.

Honoraria are paid for meetings attended in person or by telephone. For a one day meeting, such as a launch meeting, the honorarium is £150 and £75 for a half day meeting. For teleconferences, the honorarium is £50 per meeting. This is in line with Department of Health guidance.

You can also request up to £50 per meeting to cover care costs such as costs for a childminder, or to pay someone to care for an elderly relative.

Please make sure you talk to the Trial Manager or Data Manager to get an expenses claim form and to find out what you need to do to claim expenses.

**5.** **Who can I talk to if I have questions or concerns about the trial I am involved in?**

Many TMGs offer their consumer member a mentor – someone the consumer can contact if they have questions or concerns. If you do not have a mentor, or if you cannot get hold of them, or you are not satisfied with their answer, you have several options:

***The Trial Manager*** can help you with any queries, or can point you in the direction of someone who can help, if they cannot. You could also speak with the ***Project Lead***. You might prefer to talk with ***the Chief Investigator*** if you have concerns about the running of the trial. In the unlikely event that your concerns are not addressed by any of these people, and you are worried about the conduct of the trial, you can contact Max Parmar, the Director of the MRC CTU. Finally, if you are still not happy you can contact the trial sponsor, which is usually the Medical Research Council. Information about the sponsor will be on the trial protocol.

**6. Other groups and organisations connected to the TMG**

**6.1 The Trial Steering Committee**

As well as a TMG, we also have Trial Steering Committees (TSCs). The TSCs make sure that trials are conducted properly and that they follow the guidance laid out in the Good Clinical Practice guidance.

The TSC has membership from the TMG plus independent members. Within the MRC CTU Cancer Group, there are two TSCs that cover a number of topic areas; only a couple of trials have their own TSC. So for example we have a TSC which covers all of our urology trials.

The role of the TSC is to provide overall supervision for the trial. It agrees the protocol and meets at least once every year to review the progress of the trial. The ultimate decision about whether a trial carries on or not lies with the TSC.

**6.2 The Independent Data Monitoring Committee**

The Independent Data Monitoring Committee (IDMC) looks at how the trial is progressing. The people on the IDMC must be independent of the trial if the IDMC is to be independent. They are the only group who sees the confidential, accumulating data to the trial i.e. the interim results. They then comment on these results and make any recommendations they think necessary.

They can also ensure that the trial is safe. If the IDMC thinks that a trial should not continue, perhaps because people are experiencing serious side effects that were not expected, this committee can recommend that a trial is stopped.

Sometimes a trial is stopped for positive reasons. It may have interim results that are so good that carrying on is not fair to people who are not getting the research treatment. Alternatively a trial elsewhere may report results that mean this trial is no longer needed.

The IDMC will usually meet 6-12 months after the trial begins and then decide the frequency of future meetings.

**6.3 The trial sponsor**

Guidance produced by the English Department of Health requires every research project that is taking place within the NHS to have a sponsor. This guidance is called the Research Governance Framework for Health and Social Care. There is similar guidance covering Wales, Scotland and Northern Ireland. The trial sponsor is responsible for ensuring that there are proper arrangements in place to plan, manage and monitor the trial. Under the legislation that covers the conduct of clinical trials in the UK the sponsor is also responsible for ensuring that finance is in place for the trial. For most MRC CTU trials, the sponsor is the Medical Research Council. The legislation is the Medicines for Human Use (Clinical Trials) Regulations 2004.

**6.4 The Medicines and Healthcare Products Regulatory Agency**

Under the Medicines for Human Use (Clinical Trials) Regulations 2004, sponsors (see 6.3 above) must have approval from the Medicines and Healthcare Products Regulatory Agency (MHRA) to run clinical trials that involve any medicinal products. Without this approval, a trial cannot take place. Researchers must tell the MHRA if trial participants experience unexpected and/or serious adverse reactions during the trial. Such incidents are called suspected unexpected serious adverse reactions (SUSARS) and serious adverse reactions (SARS).

**6.5 Ethics Committees (or Research Ethics Committees)**

If a trial is taking place anywhere in the NHS (for example at a hospital or GP surgery, or in the community through a district nurse), it must be approved by an ethics committee. This committee includes members of the public, researchers and health care professionals. A trial cannot go ahead if an ethics committee has not approved it. The protocol for a trial cannot be changed without the approval of the ethics committee. Researchers must tell the ethics committee if trial participants experience unexpected and serious side effects during the trial (see also 6.4 above).

**7. Glossary of terms**

**We explain below the most common technical terms that are likely to be used in a TMG discussion. We have not included any clinical terms (for example clinical terms related to cancer). For a glossary of clinical terms, we recommend Cancer Research UK’s Cancer Help website –** [**http://www.cancerhelp.org.uk/glossary.asp**](http://www.cancerhelp.org.uk/glossary.asp)

**If a word is in red, it means that this word is also included separately in our glossary. Please note that some of the terms below are statistical terms, and we recognise that that is a specialised topic in itself.**

**If jargon is used during a TMG meeting, please ask for an explanation. The chances are that you will not be the only person who does not understand. And please let us know if you think other words should be added to this glossary.**

| **Term** | **Explanation** |
| --- | --- |
| **Alternative Hypothesis** | The hypothesis that there IS a difference between the treatments being studied. This might be that one is better than the other or that one is no worse. |
| **Adjuvant chemotherapy** | This is chemotherapy given in addition to the main treatment for cancer. For example, doctors might treat someone with chemotherapy as well as with surgery to try to get rid of a tumour. |
| **Adverse events** | Adverse events are undesired effects that may or may not be related to a treatment. For example, if you are given a drug to treat an illness and you become sick (e.g. dizziness, stomach ache or a rash), this would be described as an adverse event. If your sickness is caused by the drug, this would be called a side effect. Clinical trials will often look at both short- and long-term adverse events related to a treatment. Some adverse events may be serious and need to be reported to regulatory authorities (in the UK this is the [MHRA](http://www.mhra.gov.uk/index.htm)). |
| **Bias** | When people’s individual opinions lead to incorrect conclusions about the effects of treatment, this is bias. It is very important to avoid bias in health research, as it can distort the results and could lead to unsafe or inefficient treatments being licensed for use, or useful treatments being overlooked. Researchers try to avoid bias by using randomisation and where appropriate by ‘blinding’ those assessing the results of treatments, or those receiving the treatment. |
| **Blinding/ double blinding** | Blinding means that whoever is assessing the effects of treatment will not know which treatment the person has received. This helps to prevent bias. Sometimes patients will assess the effects of treatment, sometimes the researcher will, and sometimes a researcher who is independent of the trial will carry out this assessment. Blinding means that some or all of these people may be kept unaware of which treatment the person has received.  In a double blind trial, neither patients nor the researchers running the trial will know which treatment patients are receiving. The aim is to avoid any hopes and expectations about the treatment from patients or researchers, which could influence the way the improvements and side effects are assessed.  It is not always possible to avoid researchers and participants knowing which treatment they are having. For example, the trial may be comparing an injection with a tablet. If the researcher knows which treatment you are receiving, it may be necessary for another researcher, who has not been involved in conducting the trial, to assess the impact of the different treatments. |
| **Clinical trials** | Clinical trials are research studies involving patients or participants, which compare a new or different type of treatment with the best treatment currently available. They assess whether the new or different treatment is safe, effective and any better than what already exists. No matter how promising a new treatment may appear during tests in a laboratory, it must go through clinical trials before its benefits and risks can really be known. |
| **Cohort study** | A cohort study is a type of observational study. Researchers observe what happens to a group of people who have a similar characteristic – e.g. they all smoke. |
| **Compliance/adherence** | Compliance (or adherence) in the context of a clinical trial means sticking to your treatment. So for example, if you are asked to take tablets as part of a trial, it is about taking the right amount of tablets in the right way and at the right time, and finishing the course. |
| **Confidence interval (CI)** | The range in which it is thought that the real answer will be found with a given degree of certainty. For example a 95% confidence interval of 0.1−0.5 would mean that we are 95% confidence that the real answer is between 0.1 and 0.5. |
| **Controlled trial** | A controlled trial compares two groups of people: an experimental group who receive the new treatment and a control group, who receive the standard (or usual) treatment or a placebo. The control group allows the researchers to see whether the treatment they are testing is any more or less effective than the standard or usual treatment. |
| **Cross-over trials** | If you take part in a crossover trial, your treatment will change partway through the trial. So, for example, if a trial is comparing the effectiveness of 2 different sorts of exercise to help combat depression, you might take part in exercise A for the first part of the trial and then exercise B for the second, then perhaps back to A again - and so on. You cross over from one treatment group to the other, and comparisons are then made between how well you felt during the different periods. Often there will be several cross-overs in a cross-over trial |
| **Data Monitoring Committee** | Most large scale trials have an Independent Data Monitoring Committee that follows the progress of the trial and makes sure it is safe and ethical to continue. The people on the data monitoring committee are independent of the researchers running the trial. If they think that participants are experiencing serious or unexpected side effects, or if evidence has emerged that one of the treatments being compared is clearly better than the others, they can advise that a trial is stopped. |
| **Disease free survival** | The time from when a patient enters a study to the time when they have disease or they die. |
| **Efficacy** | The effectiveness of one treatment compared to another treatment |
| **Eligibility criteria** | All trials have guidelines about who can take part. These are called 'eligibility criteria', consisting of inclusion criteria and exclusion criteria. For example, the eligibility criteria for a trial looking at radiotherapy for breast cancer might say that the only people who can take part are people who have never had radiotherapy before. |
| **Endpoint** | A study outcome measure. In effect the endpoint is the main result that the trial is trying to find. |
| **Epidemiological study** | An epidemiological study looks at how often diseases occur in different groups of people and why. For example, an epidemiological study in HIV/AIDS might ask:   - How common is HIV in a particular part of the world? - Who has HIV? (For example, do more women than men have HIV? What age are the people who have HIV?) - How did they get HIV? (For example, was it through unprotected sex? Or from their mother when she gave birth?) |
| **Equivalence trial** | An equivalence trial is a study that aims to show that two treatments are the same, in terms of how well they work (their efficacy). |
| **Ethics** | Ethics are a set of principles that guide researchers who are carrying out research with people, for example, “First do no harm.” Ethical principles are designed to protect the safety, dignity, rights and well-being of research participants. They include the requirement to ask each individual to give their informed consent to take part in a research project. |
| **Ethics Committees** | The job of an ethics committee is to make sure that all research projects respect the dignity, rights, safety and well-being of the people who take part. In the UK, ethics committee approval is needed for all research that takes place within the NHS. Ethics committee members include researchers and health care professionals as well as members of the public. |
| **Evidence base** | An evidence base is a collection of all the research data currently available about a health or social care topic, such as how well a treatment or a service works. This evidence is used by health and social care professionals to make decisions about the services that they provide and what care or treatment they are able to offer people who use services. |
| **Exclusion criteria** | Exclusion criteria determine who won’t be able to join a trial – for example, many trials exclude women who are pregnant, or who may become pregnant. This avoids any possible danger to a baby. Trials may also exclude people who are taking a drug that interacts with the treatment being studied. |
| **Factorial design** | A study that looks at the effects of more than one treatment (or factor) all at the same time. |
| **Good clinical practice, or GCP** | The law in the UK states that all clinical trials that involve humans must comply with a set of requirements for good clinical practice. This law is called the Medicines for Human Use (Clinical Trials) Amendment Regulations 2004. It aims to ensure that clinical trials are ethical and of high quality. A government agency called the Medicines and Healthcare Products Regulatory Agency (MHRA) is responsible for checking that all trials in the UK comply with this law. |
| **Hazard ratio** | This is the risk of an event in the research/treatment arm relative to the control arm and is used to help compare the outcomes in the two groups. |
| **Health economics** | In some clinical trials, it can be important to compare how much different treatments cost, as well as how well they work. This can be particularly important when two (or more) treatments are equally effective, but where one costs a lot more than the other.  The gathering of this information about costs is called health economics. Health economic evaluation gives researchers and policymakers a way to think about health benefits, and costs. This enables them to try to get the best health gain for the most people, within a limited budget.  For example, economic costs involved in treating cancer nationally include the cost of treatment, care and recovery, as well as the costs of prevention, research and training of healthcare personnel. Other costs include the economic costs of illness and premature death, the loss of economic productivity, decreases in the productivity of family members, and welfare and health insurance expenditure. |
| **Hypothesis** | An unproven theory that is assessed by a trial/study. |
| **Inclusion criteria** | Inclusion criteria help researchers decide who can join a trial. For example, some trials include only people of a certain age, or at a particular stage in their illness. You may have to have a medical examination before a trial (e.g. a blood test) to assess whether you are suitable to take part. (See also eligibility criteria and exclusion criteria) |
| **Informed consent** | A patient cannot be entered into a trial without signing a form saying that they have given their informed consent. There are exceptional trials, which are set in emergency situations when researchers may not be able to gain consent from patients (for example, in a trial about head injuries in crashes, the patient would be unconscious when admitted to hospital). If a patient signs the form, they are saying that they believe they have been given all the important facts about a trial, they understand them and that they have decided to take part in the trial of their own free will. Even after giving informed consent, they are still free to withdraw from the trial at any time without giving a reason and without it having an impact on their healthcare. |
| **Intention to treat (ITT)** | Used when analysing data from randomised controlled trials. It is mostly used in superiority trials. When analysing the data all participants are included according to their allocated group. Researchers assume that the patient has taken/received the intended treatment when in fact they may not have done. This helps to minimise bias in the difference seen between treatment groups and should give ‘conservative’ results so as not to overestimate the difference between the two treatments. For example, if a patient in a clinical trial is given a drug, but doesn’t take it and throws it away instead, the researchers will assume that the patient took the drug if they are using intention to treat to analyse the data. |
| **Interim analysis** | A formal analysis conducted during a trial to check the accumulation of data. Usually reviewed by the IDMC |
| **Intervention** | Within the context of healthcare, an intervention is something that is given to a patient as a treatment, i.e. to “intervene” to stop or reverse the progress of an illness or condition. For example, giving a drug is an intervention. Counselling is also an intervention. |
| **IPD meta-analysis** | See meta-analysis |
| **Kaplan Meier curve** | A graph that displays the probability of a defined outcome measure in a group of patients over a given time period. The curve on this graph enables researchers to think about what would have happened to patients who leave a study before the specified outcome has been observed. The outcome will be a time to event outcome where the event is disease, progression or death. |
| **Log rank test** | Used to compare a defined outcome in two or more treatment groups. The outcome will be a time to event outcome where the event is disease, progression or death. |
| **Mann Whitney test** | Used to compare data from two groups (usually by comparing the medians). |
| **Mean** | The most common definition for average. It is the sum (i.e. the total) of a group of numbers divided by how many there are. For example the mean in this group of numbers  5, 8, 11, 14, 21, 25, 28 is 16 (5+8+11+14+21+25+28=112, divided by 7 = 16) |
| **Median** | It is the middle number in a group of numbers arranged in order of size. For example, the median of this group of numbers: 3, 8, 9, 13, 19, 24, 26 is 13, because it is the number in the middle when these numbers are arranged in order of size. 5, 8, 11, 14, 21, 25, 28 is 14. Note that the median is not necessarily the mean. |
| **Meta-analysis** | A meta-analysis involves a researcher bringing together the numerical results of previous research (usually randomised trials) about one particular treatment.  A meta-analysis can be important because it allows us to pick up small differences between treatments. These differences can be very hard to spot, so trials need to include large numbers of patients to pick these up. Many trials are not big enough, so we cannot be sure whether any differences that we find are because of differences between the treatments or just due to chance. By bringing together the results of all trials of a particular treatment in a meta- analysis, we can look at the experience of many more patients. This gives a more reliable and accurate measurement of the effect of the treatment and a good chance of seeing which treatments are best.  In an individual patient data (IPD) meta-analysis, the researchers go back and look at the records for each patient who took part in a trial (these are anonymised, so the researchers don’t know patients’ names). Then they bring these individual results together. This makes the results of the meta-analysis more reliable and enables researchers to look at how treatments have worked in different groups of patients, e.g. age group or sex. |
| **Methodology** | Methodology describes how research is done – it covers how information is collected and analysed as well as why a particular method has been chosen.  At the MRC CTU, methodological research (research about how to do research in the best possible way) helps us to improve how we carry out trials and other types of research. |
| **MHRA (Medicines and Healthcare Products Regulatory Agency)** | The MHRA is the government agency which is responsible for ensuring that medicines and medical devices work, and are acceptably safe. |
| **Minimisation** | Another method of allocating participants to different treatments to ensure balance between treatments and a good balance within certain groups of patients e.g. age, Sex, Tumour Type. It does this by allocating the next patient into a trial, with a probability of more than 50% to a treatment that would minimise the imbalance between groups. |
| **Multicentre trial** | A trial which is carried out across a number of hospitals or clinics, which may be in the UK and/or other countries. |
| **Neoadjuvant treatment** | A neoadjuvant treatment is a treatment that is given before the main or primary treatment. For example, neoadjuvant chemotherapy is chemotherapy which is given before surgery. The aim is usually to reduce the size of a tumour, to make it easier to remove. |
| **Non-inferiority trial** | A study which aims to show that one treatment is no worse than another. For example, a clinical trial might test if it would be appropriate to use a new treatment, which is cheaper than the existing treatment, works as well as the existing treatment. This is important because it can save the NHS money. |
| **Observational study** | In an observational study, researchers observe the people who are taking part in the study. They do not offer a treatment as part of the research. For example the MRC CTU CHIPS study is looking at what treatment HIV-infected children receive. |
| **Open trial** | An open trial has different meanings. The most common is the first one below:   - A trial that is still recruiting people. When a trial is closed, it stops recruiting people and the researchers collect and analyse the results, ready for publication. - A trial where the researcher and the participant know which treatment they are receiving – they are not blinded (see blinding). This is usually called an open label trial. |
| **Open label trials** | In an open label trial, both the patient and the doctor will know which treatment you are receiving. This is the opposite of a double-blind trial (see blinding). |
| **Outcome** | Outcomes are what happen as a result of a treatment, or the effects of a treatment. For example an outcome might be that your blood pressure is reduced as a result of taking tablets prescribed by the doctor. |
| **Outcome measures** | These are measurements of the effects of a treatment. They might include physical measurements - for example measuring blood pressure, or psychological measurements such as measuring people’s sense of well-being. So if someone takes part in research, they may be asked questions, or may be asked to have extra tests to assess how well the treatment or service has worked. |
| **Overall survival** | The time from when a patient enters a study to when they die. |
| **Per protocol analysis** | Used to assess the effect of a treatment using only those patients who complied with the protocol treatments. This is a different way of looking at data to intention to treat (ITT) analysis. It is mostly used in non-inferiority trials where the researchers are looking to see if the alternative treatment is no worse than the control. In this situation an ITT analysis may make the treatments appear too similar. We would not want to underestimate any difference in the treatments, as we do not want to say a treatment is no worse than another when in fact it is. |
| **Phase I trials** | Trials of new drugs go through a number of phases. Phase I trials aim to test the safety of a new treatment and work out the optimal dose. This will include looking at side effects of a treatment – for example, does it make people sick, raise their blood pressure etc? Phase I trials involve only a small number of people, who in most clinical trials are healthy volunteers, although in Phase 1 cancer drugs trials the volunteers are usually terminally-ill patients. |
| **Phase II trials** | Phase II trials test a new treatment in a larger group of people who usually have the disease for which the treatment is to be used, to see whether the treatment is effective, at least in the short term. Usually a few hundred people are involved at this stage. Phase II trials also look at safety. |
| **Phase III trials** | At the CTU, most of the trials we run are phase III trials. Phase III trials test the new treatment in a larger group of people. They compare the new treatment with the treatment currently in use, or occasionally with a placebo. These trials look at how well the new treatment works, and at any side effects it may cause.  Treatments only move into a phase III clinical trial if phases I and II have been successful.  In phase III trials, and sometimes in phase II trials, patients are usually randomised to receive the new treatment, the current treatment or sometimes a placebo.  Often several thousands of patients will be involved in a phase III trial. They may use different hospitals and live in different countries. At the MRC CTU, we run phase III trials across Europe, parts of Africa and the USA.  Phase III trials often need to involve large numbers of people because researchers usually need to be able to measure quite small differences between treatments. The smaller the expected advantage of one treatment over another, the more people will be needed to take part in a trial. |
| **Phase IV trials** | Phase IV trials usually look at the long term risks and benefits of a drug or treatment. They look at drugs which have already been approved for marketing and how the treatment is working once it is in general use. |
| **Pilot study** | A small preliminary study used to explore the practicalities of a larger trial/study or to provide some information to help better plan a larger trial. Such studies have the same rules and processes as larger trials. |
| **Placebo** | A placebo is a fake or dummy treatment that is designed to be harmless and to have no effect. It allows researchers to eliminate the placebo effect. The placebo effect is a psychological response where people feel better because they think they have received a treatment, and not because the treatment has a specific effect on their condition. By comparing people’s responses to the placebo and to the treatment being assessed, researchers can tell whether the treatment is having any real benefit. |
| **Power** | The probability of detecting a difference in the efficacy between two drugs. |
| **Power calculation** | Used to determine the sample size (i.e. the number of participants) needed in a randomised controlled trial to ensure that the statistical calculations when analysing the data are sensitive and accurate, and that the results will be valid statistically. |
| **Predictive variable** | A variable that predicts an outcome. |
| **Probability** | The chance that something will happen. Statisticians look at probability by using numbers between 0 and 1. 0 means something cannot happen, and 1 means something is certain to happen. So a probability of 0.9 means that something is almost certain to happen. |
| **Prognostic factor** | A prognostic factor is a condition or characteristic that can be used to estimate the outcome of a disease, or the chance of a disease coming back. So for example the size of a tumour might be a prognostic factor. |
| **Progression free survival** | The time from entry into a study to a progression (e.g. a tumour getting bigger or new symptoms developing) or death. |
| **Protocol** | A protocol is the plan for a piece of research. It usually includes information about:   - What question the research is asking and its importance/relevance - The background and context of the research, including what other research has been done before - How many people will be involved - Who can take part - The research method - What will happen to the results and how they will be publicised   A protocol describes in detail what the researchers will do during the research. In a clinical trial this includes all the medical and other staff involved in delivering the trial in hospitals and/or elsewhere. Usually, substantial changes cannot be made without going back to a regulatory authority or research ethics committee for approval. |
| **P-value** | The probability that the results seen could have occurred by chance. It has values 0 to 1. 0 means something cannot happen, and 1 means something is certain to happen. So a p-value of 0.05 means that the results are very unlikely to have occurred by chance. |
| **Quality of life (QoL)** | As well as measuring the physical effects of a treatment (for example changes to blood pressure), many trials now try to assess the impact of treatments on people’s quality of life. For example, a ‘quality of life’ (QoL) study might ask about:   - The patient’s mood and general sense of well-being - Whether they feel more tired than usual - Whether they are managing to do more things than before - Whether their sleep patterns have changed   Many QoL surveys have standard questions and common wording and are used across Europe and in the USA and many other countries. These can then lead on to other studies, for example, comparing long-term fatigue in chemotherapy patients. |
| **Randomisation** | If a patient takes part in a randomised controlled trial, they will be randomly allocated one of the treatments being compared. The decision about which treatment they’ll receive within the trial is random – or based on chance. A computer will decide which treatment they’ll receive, not themselves or the doctor. This is called randomisation.  Randomisation ensures that the groups of people in a trial are as similar as possible, except for the treatment they receive. This is important because it means that researchers can be sure that any differences between the results from the groups are due only to the treatment.  Randomisation is also the best way of ensuring that the results of trials are not biased. For example, if a doctor chose which treatment a patient should receive as part of a trial, she or he might give the new treatment to sicker patients, or to younger patients. This would make the results of a trial unreliable. Randomisation helps prevent this kind of bias. |
| **Randomised controlled trials** | Many clinical trials are randomised controlled trials (RCTs). Clinical trials aim to make a fair comparison between a new treatment and the current treatment on offer, or between two (or more) existing treatments, to see which one works best.  If you take part in a randomised controlled trial, in most cases, you will have an equal chance of receiving any of the treatments being compared. This however, may be different depending on the question being answered by the trial. The decision about which treatment you’ll receive is random – or based on chance. A computer will decide which treatment you’ll receive, not you or the doctor. This is called randomisation. |
| **Sample size** | The number of patients required in a study to make a statistically significant result likely in a given period This is also described as giving the study sufficient power. |
| **Side effects** | Side effects are undesired effects that are related to a treatment. For example, if you are given a drug to treat an illness and it makes you sick (e.g. dizziness, stomach ache or a rash), this would be described as a side effect. Clinical trials will often look at short- and long-term side effects related to a treatment. Some side effects may be serious and need to be reported to regulatory authorities (in the UK this is the [MHRA](http://www.mhra.gov.uk/index.htm)) |
| **Standard deviation** | A standard deviation shows the spread of the data, usually presented with the mean. |
| **Statistical significance** | A result that is unlikely to have occurred by chance. |
| **Statistical analysis plan** | A detailed description of the planned statistical analysis. |
| **Stratification** | A process of grouping data according to a common characteristic – for example age or weight. The process is carried out to allow for any differences within the common characteristic. For example in age, the difference between patients under 25 years and over 75 years old. |
| **Superiority trial** | A superiority trial aims to show that a new treatment is better than the standard treatment (or better than a placebo if there is no standard treatment) |
| **Systematic review** | Systematic reviews aim to bring together the results of all studies that have been carried out around the world to address a particular research question. They provide a comprehensive and unbiased summary of the research.  For example, one clinical trial may not give a clear answer about the effectiveness of a treatment. This might be because the difference between the treatments being assessed was very small, or because only a small number of people took part in the trial. So systematic reviews are used to bring the results of a number of similar trials together, to piece together and assess the quality of all of the evidence. Combining the results from a number of trials may give a clearer picture. When researchers look at the numerical results of these trials and compare them, this is called a meta-analysis. |
| **Type I error (alpha)** | Here we find a treatment effect when in fact there is not one. This is sometime referred to as a ‘false positive’. |
| **Type II error (beta)** | Here we fail to find a treatment effect when in fact there is one. This is sometimes referred to as a ‘false negative’. |
| **Variance** | This is how much a set of values varies (this is the standard deviation multiplied by itself). |
