# Supplementary material for: Involvement of consumers in studies run by the Medical Research Council Clinical Trials Unit: Results of a survey
Source: Trials. 2012 Jan 13;13:9. doi: 10.1186/1745-6215-13-9 (PMC3398265; doi:10.1186/1745-6215-13-9)
Supplement: Additional file 2 — Semi-structured Survey [file 1745-6215-13-9-S2.PDF]

MRC CTU Consumer Involvement Survey

1. Introduction

This is a survey of consumer involvement in MRC CTU research. It should take around 10 minutes to complete. We are seeking information on consumer involvement for all studies included in Appendix 1, 2009 CTU Progress Report (January 2003-January 2009).
Using these published, completed and ongoing studies we aim to:
• assess the level of consumer activity across the scope of CTU research
• provide guidance to researchers within the CTU regarding consumer involvement
• produce evidence of the impact of involving consumers on the outcomes of research

For the purpose of this survey, we define consumers as being patients, carers or family members, health service users, patient representatives or members of groups or organisations that represent those affected by the condition being researched. Consumer involvement in this context is defined as active involvement of consumers as partners in the research process and not as subjects of that research, e.g. a patient or carer as a member of the trial management group; involvement of a patient organisation in the planning of a trial or writing patient information sheets, etc.

Consumer involvement is one of the aims of the MRC CTU and we hope that the outcome of this survey will:
• encourage wider involvement of consumers throughout the CTU
• inform CTU staff of examples of best practice in consumer involvement
• lead to more effective involvement across the Unit
• provide some evidence of the impact of consumer involvement in medical research to the wider research community

We aim to analyse and publish the results of this survey. For any queries please contact cv@ctu.mrc.ac.uk

2. About the study

* 1. Please supply the title of study / study acronym

* 2. What is the disease setting for this study?

- Cancer
- HIV
- Other (please specify)

MRC CTU Consumer Involvement Survey

3. Cancer staff only

* 3. Are you aware of the guidance document available on involving consumers in your trials?

- ◯ Yes
- ◯ No

4. About the Cancer Guidance

* 4. (For cancer staff only) Have you used the guidance?

- ◯ Yes
- ◯ No

MRC CTU Consumer Involvement Survey

5. About the guidance

*** 5. Do you have any comments on the guidance?**

- [ ] It was useful
- [ ] It helped to answer my queries
- [ ] There was something missing
- [ ] It could be improved
- [ ] Other

Please provide details

6. Reasons for not using the guidance

* 6. What was the reason that you did not use the guidance?

- [ ] I read the guidance but I did not find it helpful
- [ ] It did not seem to answer my query
- [ ] Other reasons

Please supply further details

7. About the study

* 7. What is the type of study?

- Randomised Controlled trial
- Non-Randomised trial
- Observational study
- Epidemiological study
- Meta-analysis
- Other (please specify)

* 8. Was there any consumer involvement in this study?

For the purpose of this survey, we define consumers as being patients, carers or family members, health service users, patient representatives or members of groups or organisations that represent those affected by the condition being researched. Consumer involvement in this context is defined as active involvement of consumers as partners in the research process and not as subjects of that research, e.g. a patient or carer as a member of the trial management group; involvement of a patient organisation in the planning of a trial or writing patient information sheet, etc

- Yes
- No
- Don't know

If you don't know, please explain why

9. If you tried to involve consumers, how did you go about recruiting them and getting them involved in this trial?

- [ ] The consumers were already known to me
- [ ] The consumers were already known to one of the trial clinicians
- [ ] I asked the trial clinicians to recommend patients who may be interested
- [ ] I contacted a patient organisation / a consumer invovlement organisation (e.g. INVOLVE, NCRI Consumer Liaison Group etc) to help identify consumers who may be interested
- [ ] I advertised the study with groups that might be able to help reach patients

Other (please specify)

8. Reasons why inappropriate

* 10. If you considered involving consumers, but did not why did you feel it was not appropriate?

9. About the consumer invovlement

* 11. What was the motivation for involving consumers in the study? (please select all those appropriate)

- [ ] Requirement of research funders
- [ ] Requirement of the prinicipal investigator or project lead
- [ ] Specific issue to be addressed
- [ ] I have involved consumers previously
- [ ] A consumer offered their services
- [ ] Thought it was a good idea / the right thing to do
- [ ] Other (please specify)

* 12. How many consumers were involved in this study?

* 13. What specific activities did the consumers undertake (please select all those appropriate)

- [ ] Identifying topics for research
- [ ] Priority setting
- [ ] Funding applications
- [ ] Developing the protocol
- [ ] Writing / commenting on participant information leaflets
- [ ] Membership of a management/steering/advisory group
- [ ] Promoting the trial to encourage recruitment
- [ ] Reviewing / interpreting the results
- [ ] Disseminating the results
- [ ] Other (please specify)

* 14. Were there any benefits of involving consumers in this study?

- ◯ No – involving consumers made no difference to this study
- ◯ Yes (please provide examples)

* 15. Were there any problems of involving consumers in this study?

- ◯ No – involving consumers made no difference to this study
- ◯ Yes - please provide examples

* 16. Did involving consumers have a direct impact on this study?

- ◯ No – consumers had no impact on this study
- ◯ Yes - please provide examples

* 17. Did involving consumers in this study have an impact on you as a researcher?

- ◯ No – there was no impact on me from involving consumers
- ◯ Yes - please provide examples

* 18. Has involving consumers in this study influenced your decision to involve consumers in subsequent research studies?

- ◯ Yes – I plan to involve consumers in future studies
- ◯ Yes – I will not be involving consumers in future studies
- ◯ No – my ideas on consumer involvement have not changed
- ◯ Don't know – please give a reason

19. Did you receive any training or support for involving consumers in this study?

- [ ] Yes - I attended a training course (please provide details below)
- [ ] Yes - I received advice / support on consumer invovlement from a colleague
- [ ] No - I did not receive any training but I used information produced by consumer involvement organisations (e.g. INVOLVE) to help me
- [ ] No - I did not receive any support or training

Please supply further details

* 20. Is there anything that could be done within CTU to help you to include consumers in trials that you run in the future? (please select all those appropriate)

- [ ] Training
- [ ] Support / mentoring
- [ ] Guidelines
- [ ] Other – please provide details

* 21. Was training and support provided for consumers that you worked with? (Please tick as appropriate)

- [ ] They attended a training course (please provide details)
- [ ] We developed training "in house" (please provide details)
- [ ] They were supported throughout the study (please provide details)
- [ ] They had a mentor
- [ ] The consumers had no training or support (Please supply details of training / support that might have helped them)

10. Studies not involving consumers

* 22. Did you consider involving consumers in this study?

- ◯ Yes
- ◯ No
- ◯ Don't know– please give a reason

11. Future plans

* 23. Do you have plans to involve consumers in forthcoming trials?

- ○ Don't know - I haven't thought about it yet
- ○ Don't know - I am not involved in new studies at the moment
- ○ No - I am not going to involve consumers in my new studies
- ○ Yes - please give details

12. The survey is complete

Thank you for taking the time to complete the survey. We will be collecting the data and analysing results through November and December 2009 and hope to present the preliminary results to CTU staff in early 2010.
