# Supplementary material for: Involvement of consumers in studies run by the Medical Research Council Clinical Trials Unit: Results of a survey
Source: Trials. 2012 Jan 13;13:9. doi: 10.1186/1745-6215-13-9 (PMC3398265; doi:10.1186/1745-6215-13-9)
Supplement: Additional file 3 — Guidance document for Trial Managers [file 1745-6215-13-9-S3.DOC]

# MRC CTU Consumer Group

**Guidelines for involving consumers in**

**MRC CTU cancer trials and studies**

# 1. Who are ‘consumers’?

In this context, we are talking about people affected by cancer. These may be people who have had cancer themselves (or who still have it), as well as those who have cared for someone with cancer. It may also be people working for organisations that support people affected by cancer, or simply a lay person with an interest in cancer research.

# 2. Why involve consumers?

# Experience has shown that the involvement of consumers in trials and studies has a number of benefits, both for those conducting the research as well as those who benefit from the findings. Consumers can help researchers to:

- Ensure that trials and studies address questions that are important and relevant to patients and carers
- Refine the research question
- Develop patient centred outcome measures
- Improve the quality of patient information
- Consider any ethical issues and offer an opinion to an ethics committee
- Gain approval for a trial or study, including funding approval
- Recruit participants to a trial or study, individually or on a wider scale
- Disseminate the results of the trial or study, especially to other consumers[[1]](#footnote-2) and to consumer groups

Consumer Involvement has tangible and measurable benefits for researchers, and you should involve consumers with such benefits in mind, not simply because it is expected or required.

# 3. How to involve consumers in trials and studies

# First you need to think about the benefits you seek from consumer involvement, and then you need to consider *how* consumers can deliver those benefits. The simplest way to achieve many of the benefits in section 2 above is to involve consumers as members of the Trial Management Group (TMG). It is also a very good learning experience for consumers, as TMGs deal with all aspects of the research cycle.

Before you start recruiting consumers, you will need to develop a role description, outlining exactly what you would like your consumer(s) to do and how you would like them to do it. Try to be as specific as possible about any committee meetings, email discussions or teleconferencing, any of which may be novel experiences for consumers. Be clear about how much time this will involve, what skills or experience they need and whether they will be paid for their time (see section 6).

You should also state that training may be available (see section 7). If there is no formal training available, you will need to consider how much you can give yourself, perhaps in the form of mentoring, and whether or not this affects the skills and experience the consumers will need.

We have attached an example role description for involving consumers as appendix 1.

# 4. Finding consumers

Once you have developed a role description you can begin to recruit consumers. You should seek to involve at least two consumers on any TMG. Research has shown that involving one consumer is not as effective, as often they can feel marginalised and feel insignificant.

Involving two consumers will also allow them to try out ideas or to test their understanding on each other, it should ensure that at least one is available for any meeting or discussion, and it should produce a broad perspective of consumer/public/patient views.

There are several different sources for recruiting consumers. We have listed these below, in order of how easy and effective researchers have reported them to be.

Whichever source you use, ensure you use the role description to check that the people who are interested in being involved have the skills and experience you need – or if they have not, that you can help them to access this. This may include having a consumer experienced in patient and public involvement (PPI) work acting as a guide or mentor for other consumers and/or advising you on recruitment. We have included an example of an advert as appendix 2.

## A. The National Cancer Research Institute Consumer Liaison Group (NCRI CLG)

The Consumer Liaison Group (CLG) provides a forum for discussion and debate on PPI in the planning and organisation of cancer research in the NHS. It acts as a focal point for discussion, advice and feedback to the National Cancer Research Network (NCRN) Coordinating Centre, NCRI Clinical Studies Groups, the NCRI Board and other strategic groups of NCRI, on research issues directly affecting the consumer.

The CLG brings together people with personal experience of cancer, carers and representatives of cancer support organisations. Since its inception 11 years ago the CLG has established a reputation for working in partnership to create an open dialogue between patients and professionals. Meetings of the CLG take place 3 times a year. Members normally also attend 2/3 meetings of the Clinical Studies Group[[2]](#footnote-3) in which they have a particular interest and at any one time about 10-12 members are working with NCRI strategic groups[[3]](#footnote-4).

There are over 50 members of the NCRI CLG. Almost all are current or former patients, and all have an interest in cancer research and in particular cancer trials. They also have access to training, and some have been active in research for a decade or more. They are an ideal first point of contact when you are recruiting consumers to get actively involved in your trial.

Contact: NCRN Consumer Liaison Lead at Co-ordinating Centre in Leeds: Tel: 0113 392 7570; Email: [consumers@ncrn.org.uk](mailto:consumers@ncrn.org.uk)

## B. Macmillan Cancer Support’s Cancer Voices programme

Macmillan Cancer Support promotes the active involvement of people affected by cancer in a variety of activities. People affected by cancer can sign up for email alerts about opportunities to get involved in a particular type of activity – for example research, or activities relating to a particular type of cancer.

You can see more by looking at the Macmillan website, <http://www.macmillan.org.uk/Get_Involved/Cancer_Voices/Cancer_Voices.aspx>

You can advertise opportunities for involvement on the ‘opportunities exchange’. See –

<http://opportunities.macmillan.org.uk/p_homePage.aspx>

## C. The relevant cancer support charity

There are charities supporting people with most cancers. Try contacting the relevant charity (or charities), explaining your project and asking if they have anyone who is interested in being involved. To find the relevant charity, you can Google, or go to the Charity Commission website – <http://www.charity-commission.gov.uk/registeredcharities/first.asp>

There are likely to be several charities supporting people with any type of cancer – so you may want to ask the MRC Consumer Group for help, or read more about the charity via the Charity Commission website to see if they have an interest in research.

Independent Cancer Patients Voice is a charity, set up by cancer patients, which brings the opinions, views and experience of cancer patients, their family and carers, to the cancer research community. ICPV members have been actively involved in a number of trials from the design stage onwards. For more information see http://www.independentcancerpatientsvoice.org.uk/

*D. Using contacts*

You may find that the PI (or someone else who gets involved in planning the trial early on) has links with consumers who may be interested in getting involved. But be aware that it can be difficult for the clinician and the consumer if the consumer has been the patient of a clinician who is a member of the TMG.

## E. Approaching consumers who’ve been involved before

If the Unit has already run trials in a particularly disease, you may find that consumers have already been involved in a TMG. They may be interested in further involvement. Or if they are a member of a relevant organisation, they may be able to suggest someone else who might be interested in getting involved.

5. When to involve consumers

Involve consumers as early as possible, even if no funding is available at the planning stage (see section 6).

Involving consumers at the different stages of a trial or study

# *Planning the trial or study - before funding is available*

At this stage it is easier to involve consumers by phone or email – unless you have funds available to bring them to meetings. Include them in refining the research question, the trial/study design, developing the protocol and commenting on applications for funding (and/or supporting the application with a covering letter).

# *Once funding is available*

Ask consumers to help develop or comment on patient information sheets, to think about recruitment plans, and to help gain ethical approval. Some consumers have attended courses in writing in plain English (e.g. from the Plain English Campaign), or may have worked with cancer charities in designing patient information, and you may want to ask for this type of experience in your role description.

## Launching the trial or study

If there is a launch, involve consumers in planning this. It may be appropriate to include a quote from a consumer in any press releases – they could talk at the launch meeting about why this trial or study is important.

### **When the trial or study is under way**

Consumers should be seen as equal members of a TMG, and should be involved as you would involve any other member.

If you do intend to make significant use of teleconferencing however, raise this at an early stage in your recruitment of consumers, as some consumers can find teleconferencing with professionals quite daunting, especially if most members of the TMG are in one place but the consumers are on the end of a phone line, which may well be unavoidable but needs careful chairing.

## Analysing the results

Ensure you involve consumers in any discussions about analysis. (It is helpful to think about training to support consumers to be involved usefully at this stage – see section 7).

# *Results*

At this stage consumers can help to disseminate the results of the trial to their peers. You can also consider including a quote from them in any press releases and asking them to accompany you to any conferences that may be relevant to them.

Charities will be more likely to publicise the results of your research if there is a direct link with patients or their families, and consumers will provide the “human touch” to help your results to reach the audience most likely to benefit from your findings.

# 6. Providing a mentor for consumers

When a consumer joins a TMG, you should offer them a mentor, even if you have recruited experienced consumers. The mentor is someone that they can contact if they have any questions or concerns that they do not want to raise in the TMG meetings. The mentor should be someone with whom the consumer feels comfortable. It could be the Chief Investigator or another clinical member of the TMG or it could be the Trial Manager or Project Lead.

# 7. Paying consumers

As a very minimum, you need to ensure that consumers are not out of pocket as a result of getting involved in a trial. So you should budget for travel expenses for two consumers to:

- Have an initial meeting with you to learn more about the project and what is involved
- Attend management group meetings
- Attend launch events or dissemination events
- You should include a notional sum to cover care costs (e.g. costs for a childminder, or to pay someone to care for an elderly relative). If you haven’t already recruited consumers when you’re putting your budget together, we suggest you add £50 per meeting to cover these costs. If you involve consumers early enough, you can work out what to budget by asking the consumers whether they need to pay a carer, and if so, how much
- Take part in at least one training course (see section 7) – so budget for a training fee of £75 plus travel expenses and carer costs

You should also budget to pay consumers an honorarium or attendance allowance. Your funding application should include payment of **£150** per consumer per meeting, £75 per consumer per half-day meeting and £50 per consumer per teleconference meeting. This is in line with Department of Health guidance[[4]](#footnote-5).

# 8. Supporting consumers

When you recruit consumers, ask them about any training needs and any support they might find helpful to fulfil the role description that you have already given them.

It is good practice to phone or email consumers before management group meetings to check whether they understand the papers you have sent them, and to ask if they need any additional information.

After meetings, it is useful to contact them to ask them how they found the meeting, and whether they have any suggestions for improving how they are involved. In this way we can continue to improve our practice, as well as supporting and encouraging the consumers to become champions of our research, and of health research in general.

Consumers can sometimes access training provided by the NIHR Clinical Research Network and the NCRI CLG. For more information see <http://www.crncc.nihr.ac.uk/ppi> or contact the NCRN Consumer Liaison Lead at the Co-ordinating Centre in Leeds: tel. 0113 392 7570; email: [consumers@ncrn.org.uk](mailto:consumers@ncrn.org.uk).

# 9. Useful reading

INVOLVE supports public involvement in health research and produces a publication on involving consumers in research:

Hanley B et al: Involving the public in NHS, public health and social care research: Briefing notes for researchers.

You can order a paper copy via the INVOLVE website [www.invo.org.uk](http://www.invo.org.uk/), or download it: [http://www.invo.org.uk/pdfs/Briefing%20Note%20Final.dat.pdf](http://www.invo.org.uk/pdfs/Briefing Note Final.dat.pdf)

# Appendix 1: example role description

***These terms of reference were developed by Claire Vale of the Meta-Analysis Group. She calls the consumers who are involved in her project ‘research partners’. Please note that this document is from 2005 and some of the factual information is out of date.***

**Research Partners: terms of reference**

The Terms of Reference should act as a guide for the Research Partners. They should give you a better idea about your role in this project if you become a Research Partner. They explain what we would expect from you if you became a Research Partner, as well as what you can expect from us. The Terms of Reference can change in response to your feedback.

***1. Membership***

We hope to set up a group of 5-6 Research Partners to be involved in the project. The two requirements that we think Research Partners need are:

1. Personal experience of either radiotherapy or chemoradiation treatment for cervical cancer
2. Interest and enthusiasm about being involved in this project

You do not need to have any previous experience of research. And, you are free to leave at any time, although we hope that all Research Partners would like to be involved throughout the project. We expect that this project will take around 2 years to complete.

***2. What we can provide to Research Partners***

If you decide to become a Research Partner, we are committed to providing you with:

- An opportunity to learn about, actively contribute to and influence research
- Flexibility and choice in your level of involvement in the project
- A safe and supportive research environment
- Appropriate and relevant training
- A mentor, to act as your first point of contact for queries or difficulties
- Opportunities to meet other Research Partners to discuss the project
- Payment for attending meetings where needed

We will work with you to:

- Help you to learn about and understand the research project
- Identify research and/or administrative tasks that you can help with
- Provide access to appropriate training, where it might be helpful
- Support you in the activities that you undertake

We will provide a safe and supportive environment for your involvement, but the Research Partners group is not intended to be a Support Group. We are not able to give specific information or advice on treatments that you might have been given or may be receiving. However, we can put you in touch with groups or individuals who can provide emotional support and advice, should you want this.

***3. Key Responsibilities and Aims***

As a Research Partner, you will work with each other and members of the Meta-analysis Group to carry out the research project. You will also feedback your thoughts and experiences of being involved in the project to the Meta-analysis Group and Reference Group. We would also like two Research Partners to be members of the project Reference Group. This responsibility could easily be shared between the Research Partners. There are separate Terms of Reference available for the Reference Group members.

There are many aspects to running a research project like this one. Some of these are administrative or creative and not necessarily scientific or technical. We can discuss the possibilities with you to help you to find things that interest you and that you might like to be involved with. For example there may be opportunities to:

- Read and comment on the information that we produce about the project for women who have had or have cervical cancer
- Help us to organise small local meetings or the large Collaborators’ meeting at the end of the project
- Write short articles for the newsletter
- Help to produce and disseminate the results of the project to women who have had or have cervical cancer

You can be involved in one or more of the many aspects of this project. What you do might change as the project progresses. For example, once you have a clearer idea of the project, and have maybe taken up opportunities for training, you might take on different tasks or suggest other areas where your skills could put to good use. We will work with you to develop these opportunities.

***4. Meetings and Communications***

We can arrange to visit you or a have a telephone conversation with you so that you can find out more about project before you decide whether or not to become a Research Partner. Once 5 or 6 women have agreed to be Research Partners, we will organise a meeting at the MRC Clinical Trials Unit, or at another convenient location. We will try to keep this meeting (nd any additional meetings we have) very informal and we won’t use technical language or jargon. This meeting will give you:

- Chance to meet the other Research Partners
- Chance to meet the Meta-analysis Group members who are running this project
- Opportunity to find out more about the project and decide if you would like to be involved in it

After this first meeting, we will contact you in the way you prefer, and we can send you information in whichever format suits you best (e.g. on paper, email, large print etc). If you need to ask any questions or if you want us to explain something, you can contact us during our working hours to ask. We will do our best to answer all enquiries promptly and clearly. We might also organise other meetings of the Research Partners during the project, but we would like to keep the number of meetings to a minimum. We will try to arrange them to suit the needs of the individuals.

***5. Payment***

Where needed, we can reimburse you for travel or other expenses for attending meetings. These might include overnight accommodation or carer costs, for example. Wherever possible, we will try to pay for all travel tickets and accommodation bookings in advance. Otherwise, we will aim to reimburse expenses as quickly as possible. In addition, we are able to pay Research Partners for their time at the standard Department of Health rate (currently £138.71/day).

***6. Person Specification***

| **Attributes** | **Essential** | **Desirable** | **Not important** |
| --- | --- | --- | --- |
| Enthusiastic about the project | X |  |  |
| Committee Experience |  |  | X |
| Aware of issues that might affect women with cervical cancer |  | X |  |
| Understanding of medical/research language |  |  | X |
| Experience of chemoradiation or radiotherapy treatment for cervical cancer | X |  |  |
| Good communicator /able to express own views in a mixed group of professionals and consumers |  | X |  |

Claire Vale, Meta-analysis Group, MRC Clinical Trials Unit, London April 2005

IPD meta-analysis of chemoradiation in cervical cancer

**Appendix 2 – Adverts**

***Below are two adverts – one was placed on the Prostate Cancer Charity’s Prostate Cancer Voices website, to recruit a consumer member for the RADICALS trial. The second was developed to recruit consumers for two ICON trials:***

| **Opportunity** | ***We’re looking for a patient representative to join the Trial Management Group of an ongoing prostate cancer clinical trial*** |
| --- | --- |
| The Medical Research Council Clinical Trials Unit is running a large prostate cancer clinical trial throughout the UK and internationally. The trial, called RADICALS, is hoping to answer two important questions for men that have had surgery for prostate cancer:   - Which is the best way to use radiotherapy after surgery? - Which is the best way to use hormone treatment with any radiotherapy given after surgery?   The trial is guided by a Trial Management Group (TMG). The TMG has members to represent the perspectives of all of the stakeholders on a trial. This includes cancer doctors, surgeons, nurses, statisticians, trialists and patient representatives.  It is important that at least one member of the TMG is a patient representative so that any decisions that are made about the trial are thought about from the perspective of the patients that are on the trial or that might join the trial. The original patient representative stepped down for ill-health.  You would be asked to join TMG meetings via teleconference approximately every 6-8 weeks. Usually there are some documents to review before the teleconference. The meetings are typically 45 minutes. You would be asked to contribute your thoughts on decisions that the group makes from a patient’s point of view. You would also be asked to raise some awareness of the trial with other men affected by prostate cancer. This might be, for example, through meetings for prostate cancer support groups or highlighting the study in newsletters.  We would reimburse you for any expenses you incur and provide a small payment at the rate recommended by the Department of Health (£50 per teleconference) | |
| **Who should get involved?** | Men affected by prostate cancer that are interested and enthusiastic about being involved with this large clinical trial. It would be useful but not essential that you have had surgery for prostate cancer (radical prostatectomy) and/or experience of being in a clinical trial.  You should have access to email as most communication is via email in between teleconference meetings. |
| **Closing date for application** | 10^th^ December 2010 |
| **When will this activity take place?** | We would like you to begin joining the TMG teleconference meetings at the beginning of next year. The trial is expected to continue recruiting until 2014 and the TMG will remain in place after that time. You would be, of course, free to leave the group at any time. |
| **How to get involved** | Email RADICALS@ctu.mrc.ac.uk |
| **Where will this activity take place?** | You can dial into the teleconference meetings from home. We will provide a free-phone number for you to call. We do not expect you to join any in‑person meetings but in the unlikely event that there is a meeting, it will most likely be in London. |

**Gynaecological Cancer Patient Representatives**

**Organisation:** Medical Research Council Clinical Trials Unit (MRC CTU)

**Location:** 222 Euston Road, London, NW1 2DA

**About the Gynaecological Cancer Group at the MRC CTU:** The MRC CTU has coordinated a number of collaborative trials investigating treatment for ovarian cancer. Our current research is exploring the effectiveness of adding cediranib to chemotherapy treatment for women with relapsed ovarian cancer, in our ongoing trial called ICON6. We are also in the process of designing a new trial (ICON8) to see if it can improve survival outcome for women with newly diagnosed ovarian cancer.

**About the ICON6 trial:** ICON6 is a ongoing research study (also known as a ‘randomised controlled trial’) investigating whether we can improve the benefits of chemotherapy cancer treatment for women with platinum-sensitive relapsed ovarian cancer, by adding a new drug called cediranib (also known as Recentin and previously as AZD2171). Although there are good scientific reasons from laboratory studies why cediranib should work on ovarian cancer cells, it has not yet been tested in a large number of women with ovarian cancer. Some early clinical studies have shown that cediranib reduces the size of some ovarian tumours. This needs to be confirmed and we need to find out whether cediranib improves the effectiveness of chemotherapy, and whether continuing cediranib after chemotherapy is beneficial. The trial hopes to recruit a total of 2000 women over the next 2 years (currently we have recruited almost 180 women).

**About the ICON8 trial:** Women with newly diagnosed ovarian cancer usually have a good response to treatment with surgery and chemotherapy but the 5-year survival rate remains lower than preferred. ICON8 is a randomised trial investigating chemotherapy given weekly rather than 3-weekly with different options for timing of surgery to see if it can improve survival outcome.

These trials are coordinated by the MRC CTU and are funded by Cancer Research UK.

**Purpose of involvement:** We would like two enthusiastic and interested individuals who have personal experience of ovarian cancer (or who have close association with a person who has experience of ovarian cancer) to work in partnership with us on the existing ovarian cancer trial (ICON6) and on the new ovarian cancer trial (ICON8) due to start recruiting patients in April 2011.

By participating the patient representatives can put personal experiences to positive use by contributing to the project, influencing the way it is carried out

and making sure that the results are made widely available to other women diagnosed with ovarian cancer. We hope that working together we can:

- Aim to ask research questions that are more relevant to women with ovarian cancer
- Better reflect issues that are important to women with ovarian cancer
- Provide information that might help women in the future who have to make choices for treatment of ovarian cancer
- Involve enthusiastic people in a new research partnership
- Help patients to lean about the type of research we do

**Involvement required:** There are many aspects to running a research project like this one. Some of these are administrative or creative and not necessarily scientific or technical. We can discuss the possibilities to find things that interest you and that you would like to be involved with. For example there are opportunities to:

- Provide input into the study design
- Comment on information we produce for patients and other documentation
- Help interpret the results of the research
- Help disseminate the results to women with ovarian cancer

**Method and frequency of involvement required:** We will hold group meetings or teleconferences around twice per year. The patient representatives can also be involved via email, phone etc. and some time is needed for reading, commenting and providing feedback. Extent of involvement can be adapted to suit individuals’ time and interests.

**Venue and location:** Meetings are likely to be held at the MRC CTU, London or by teleconference call.

**Period of involvement:** Involvement will commence as soon as possible. We would like the patient representative to provide input into the study design of ICON8 before it is finalised. We anticipate that the ICON8 trial will commence in April 2011 and will continue until 2016. The ICON6 trial opened to recruitment in December 2007 and is estimated to continue until 2014. We would like the patient representatives to work with us throughout these projects and any further ovarian cancer trials that the group may be involved with in the future. The patient representatives are however free to leave at any time.

**What we can provide to Patient Representatives:** If you decide to become a Patient Representative, we are committed to providing you with:

- An opportunity to learn about, understand and actively contribute to and influence research
- Flexibility and choice in your level of involvement in the project
- Support you in the activities that you undertake
- Provide access to appropriate training, where it might be helpful
- Opportunities to meet other Patient Representatives to discuss the project
- Payment for attending meetings

**Interests/experience desired:**

- Interested and enthusiastic about contributing to this research project
- Personal experience of ovarian cancer or close association with a person who has experience of ovarian cancer
- Good communication skills
- Previous experience of research is not required

**Rate of reimbursement for travel expenses paid:** All public transport costs and mileage will be paid or reimbursed. We can also pay patient representatives for their time at the standard department of health rate of £150 per meeting, £75 per half-day meeting and £50 per teleconference meeting.

**If you are interested, please contact the Gynae team on** [**ICON8@ctu.mrc.ac.uk**](mailto:ICON8@ctu.mrc.ac.uk)**.**

1. For more information see the results of the survey of consumer involvement in MRC CTU studies – in press [↑](#footnote-ref-2)
2. NCRI Clinical Studies Groups are responsible for the development of national cancer clinical trials[.](http://ncrndev.org.uk/index.php?option=com_content&task=view&id=31&Itemid=274) They oversee existing studies, consider new research questions, develop proposals and secure funding from NCRI members and other sources, as well as providing expert advice. [↑](#footnote-ref-3)
3. NCRI Strategic Groups look at issues which cut across a range of cancers – e.g. supportive and palliative care, PET scanning. [↑](#footnote-ref-4)
4. Note these figures are correct at the time of writing (August 2011) but should be checked with INVOLVE – www.invo.org.uk [↑](#footnote-ref-5)
